# Bilateral Subclavian Steal Syndrome

**DOI:** 10.1155/2011/146267

**Published:** 2011-07-06

**Authors:** Reza Amini, Heather L. Gornik, Leslie Gilbert, Sue Whitelaw, Mehdi Shishehbor

**Affiliations:** Section of Vascular Medicine, Heart & Vascular Institute, 9500 Euclid Avenue, Cleveland Clinic, Cleveland, OH 44195, USA

## Abstract

Bilateral subclavian steal syndrome is a rare condition. It is usually due to reversal of vertebral blood flow in the setting of bilateral proximal subclavian or left subclavian plus innominate artery severe stenosis or occlusion. This finding may cause cerebral ischemia related to upper extremities exercise. We report a case of bilateral subclavian steal secondary to total occlusion of the innominate and left subclavian arteries in a patient who presented with cardiomyopathy and flow reversal in the right carotid and bilateral vertebral arteries.

## 1. Introduction

Subclavian steal syndrome was first described by Contorni in an asymptomatic patient in 1960 [1] and was subsequently associated with potential for cerebrovascular symptoms [2]. It is usually due to reversal of vertebral blood flow in the setting of proximal subclavian or innominate artery severe stenosis or occlusion. This often results in cerebral ischemia related to exercise of the upper extremities. Subclavian stenosis can be easily diagnosed by comparing bilateral brachial systolic blood pressures (>15 mmHg difference) and is an independent risk factor of overall and cardiovascular mortality [3]. Subclavian steal typically occurs only in the setting of severe stenosis or occlusion. Bilateral subclavian steal syndrome is a rare condition.

## 2. Case Report

Fifty-eight-year-old female presented to our center with progressive shortness of breath and fatigue over the past month. Shortness of breath was mainly with exertion and had been progressive. She reported sleeping on five pillows and had required oxygen therapy at night for the last 1.5 years but denied paroxysmal nocturnal dyspnea. She denied pain in the arms with exertion or lightheadedness when using her arms.

Her medical history was significant for chronic obstructive pulmonary disease, hypertension, and peptic ulcer disease. She was smoker, one pack per day for 47 years. She also had history of heavy alcohol consumption. Physical exam was significant for “normal” and equal blood pressure in arms (right arm 111/91 mmHg, left arm 104/82 mmHg). There was no distension of the jugular veins. There were bilateral carotid bruits. Cardiac examination revealed a normal rate and regular rhythm, with a grade II/VI holosystolic murmur over the apex radiating to the axilla. There was no gallop or friction rub. Lungs were clear to auscultation. Liver was palpated 4 cm below costal margin. There was mild pitting edema noted over both lower extremities. Pulses in bilateral upper extremities were diminished with normal pulses in bilateral lower extremities.

## 3. Methods

The patient was admitted to the hospital and diagnostic workup was performed, including blood workup, EKG, chest X-ray, transthoracic echocardiogram, and high-resolution B-mode carotid duplex ultrasound (Philips iU22, Phillips Electronics, Bothell, WA, USA) with color and pulsed wave doppler using L7–4 MHz linear array transducer. On the bases of these studies, the patient was subsequently referred for head and neck computed tomographic angiography (CTA) and coronary artery and arch angiography.

## 4. Results

**Figure 1:**
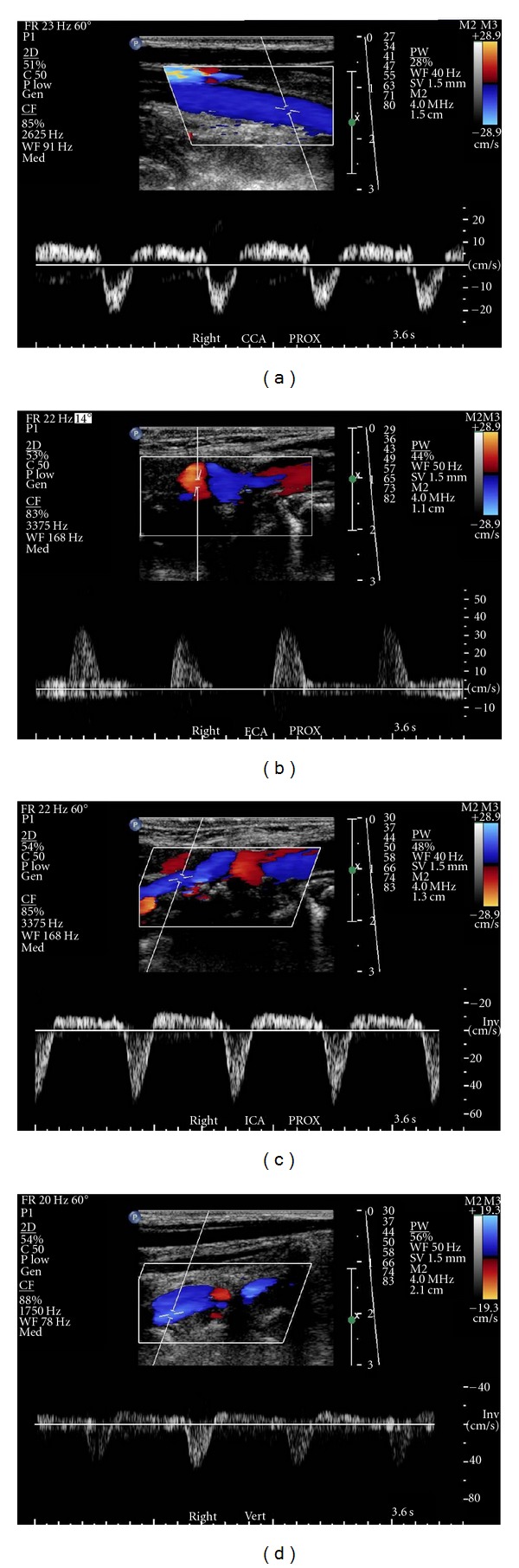
Color and pulsed-wave Doppler showing flow in right carotid and vertebral arteries. (a) To- and- fro with mainly retrograde blood flow in right common carotid artery. (b) Right external carotid artery with reversal of blood flow. (c) To- and- fro with mainly retrograde blood flow in right internal carotid artery. (d) Bidirectional and primarily reversed blood flow in right vertebral artery. Flow in the vertebral vein and the vertebral artery are in the same direction (toward the heart) and shown in blue. Note all arterial color Doppler flows are retrograde in these images.

**Figure 2:**
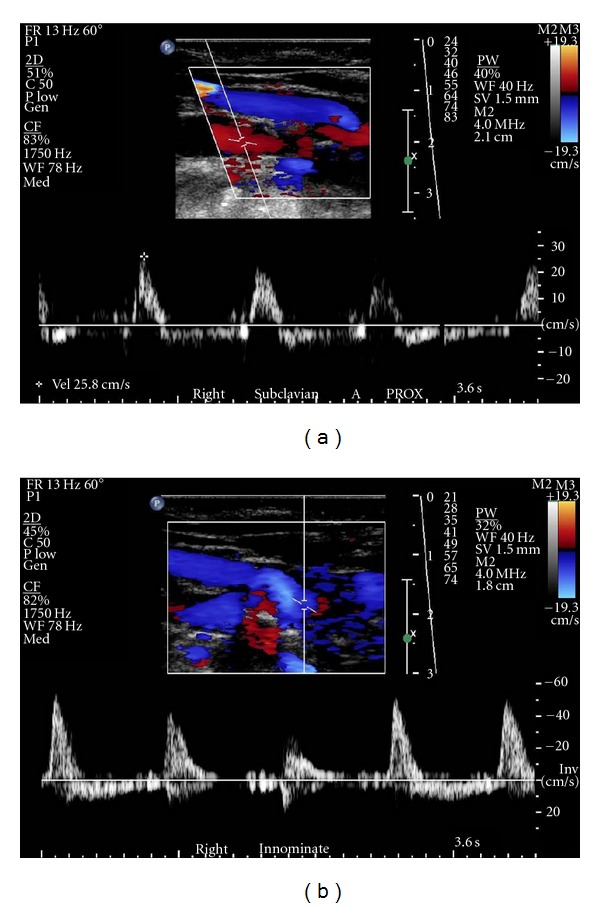
Color and pulsed-wave Doppler showing flow in right subclavian and innominate arteries. (a) Low velocity to- and- fro blood flow in the right subclavian artery. The very abnormal appearance of the spectral waveforms suggests more proximal severe stenosis. (b) To- and- fro blood flow in the innominate artery. There is spectral broadening present. Color flow image demonstrates retrograde flow in this single image.

**Figure 3:**
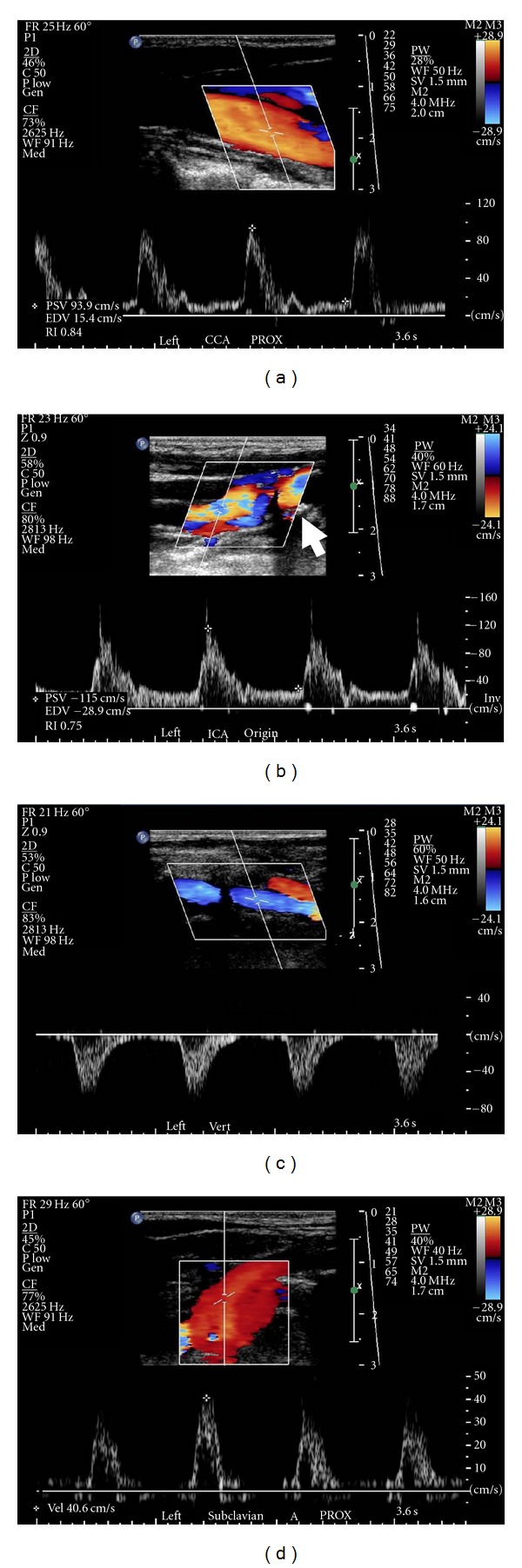
Color and pulsed-wave Doppler showing flow in left carotid, vertebral, and subclavian arteries: (a) showing proximal left common carotid artery. There is a relatively normal-appearing Doppler waveform, (b) note mild elevation in peak systolic velocity with plaque at left internal carotid artery origin (arrow). The stenosis is categorized as 40–59% in our laboratory. (c) Showing complete reversal of blood flow in left vertebral artery consistent with complete subclavian steal. (d) Low velocity blood flow with abnormal-appearing Doppler in left subclavian artery consistent with more severe subclavian stenosis proximal to this site.

**Figure 4:**
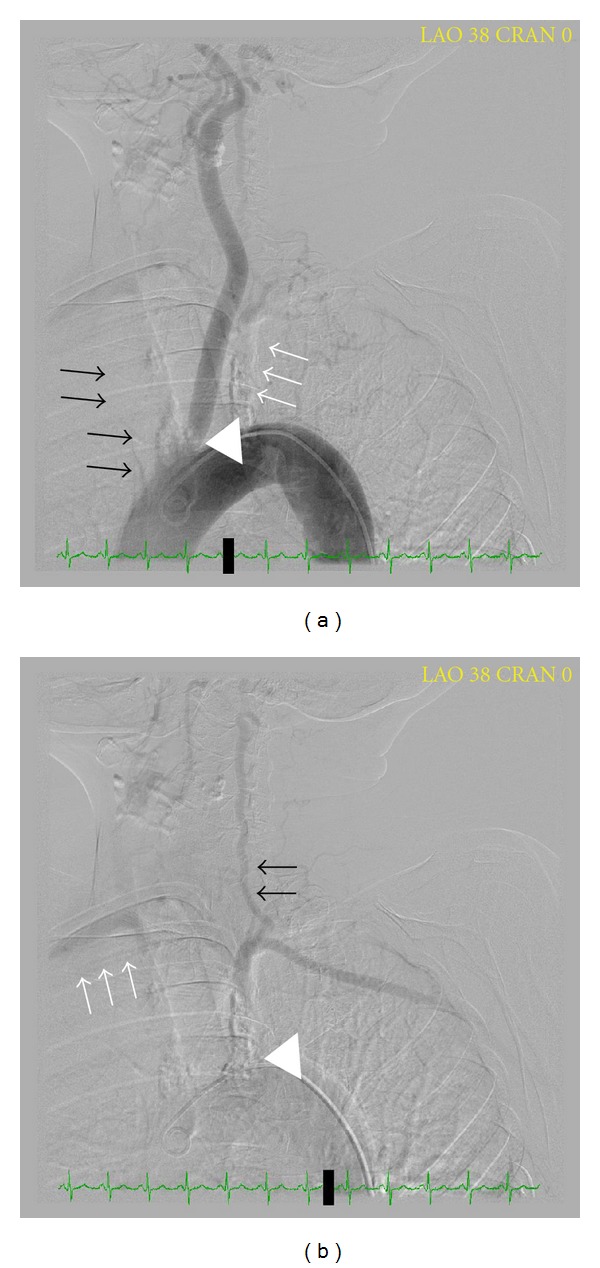
Arch aortography revealing a Type II arch. (a) Totally occluded innominate artery (black arrows) and left subclavian artery (white arrows). Note the left common carotid artery with 50% ostial stenosis (white arrow head). (b) Left subclavian artery (white arrow head) is heavily calcified and totally occluded. It is filled via retrograde flow from left vertebral artery (black arrows). Right subclavian artery (white arrows) is filled via retrograde right vertebral and carotid arteries (not seen well on this angiogram).

The electrocardiogram findings were nonspecific except for left axis deviation. Cardiac enzymes were within normal limits. B-type natriuretic peptide was 1926 pg/mL (upper limit of normal = 99), consistent with volume overload/congestive heart failure. Chest X-ray was within normal limits. The echocardiogram showed dilated left ventricle with an ejection fraction of 20% severe mitral and moderate tricuspid valvular regurgitation with right ventricular end systolic pressure of 44 mmHg. Carotid duplex ultrasound examination was performed to evaluate bilateral carotid bruits (Figures 1–3). This demonstrated reversal of flow in right external carotid artery, bidirectional (to-and-fro) flow with primary retrograde flow in the right internal carotid artery, right common carotid artery, and right vertebral artery suggesting severe innominate artery stenosis (Figures 1(a)–1(d) and 2(a)). The right subclavian artery was filled via a combination of retrograde flow from the right vertebral and common carotid arteries (supplied via retrograde flow from right external carotid artery) (Figure 2(b)). On the contralateral side, left common carotid artery had relatively normal-appearing pulsed Doppler waveform (Figure 3(a)). Left internal carotid artery color and pulsed-wave Doppler showed mildly elevated peak systolic velocity with plaque at vessel origin (Figure 3(b)). The stenosis is categorized as 40–59% in our laboratory. Left subclavian artery was filled via retrograde flow through left vertebral artery suggesting concomitant left subclavian artery stenosis (Figures 3(c) and 3(d)). Computed tomographic angiography of the head and neck was performed. There was severe arthrosclerosis of the aortic arch vessels with near total occlusion of innominate artery and heavy calcification of left subclavian artery. The patient subsequently underwent coronary and arch angiography. This revealed aortic pressure of 220/80 with minimal nonobstructive coronary artery disease. Arch angiogram plus selective arch vessel angiography revealed heavily calcified and occluded innominate artery (Figure 4(a)). The left common carotid artery had dense, heavily calcified plaque proximally with a 50% ostial stenosis (Figure 4(a)). The left subclavian artery was heavily calcified and totally occluded (Figure 4(b)). 

## 5. Discussion

Subclavian steal syndrome refers to the phenomenon of reversal of blood flow in vertebral arteries usually caused by stenosis of the subclavian artery. Subclavian steal may result in arm or cerebrovascular ischemia. Bilateral subclavian steal syndrome is a rare condition. Atherosclerosis is the most common cause of subclavian steal syndrome. Due to acute origin of left subclavian artery resulting in accelerated atherosclerosis due to turbulent blood flow, it is the most commonly affected artery (72%) versus the right subclavian artery and innominate artery which are affected less commonly (16% and 10–12%, resp.) [4, 5]. The Takayasu arteritis, giant cell arthritis, external compression of subclavian artery at the thoracic outlet, traumatic, and congenital anomalies such as anomalies of the innominate artery are among some other conditions which can cause subclavian steal physiology [6–8]. Subclavian artery stenosis is an independent risk factor for total and cardiovascular mortality after adjusting for age, gender, ethnicity, cohort of origin, cardiovascular diseases risk factors (smoking pack-years, hypertension, diabetes, total/high-density lipoprotein cholesterol ratio, and body mass index), as well as lipid lowering and antiplatelet therapies [3].

Most patients with subclavian steal are asymptomatic, and reversed vertebral flow is found incidentally during duplex ultrasound. Among symptomatic cases, they mainly present with arm ischemia symptoms including exertional pain and weakness (arm claudication), which is due to the severe subclavian stenosis. Symptoms of vertebrobasilar ischemia of brainstem (dizziness, vertigo, ataxia, disequilibrium, drop attacks, diplopia, nystagmus, graying of vision, hemianopia, bilateral visual blurring, and syncope) are uncommon and usually occur in patients with concurrent cerebrovascular lesions [9].  In some cases, reduced arterial flow resistance, during upper extremity exercise, can precipitate vertebrobasilar symptoms. Collateral blood supply and the capacity to increase collateral flow determine patients who develop neurologic symptoms. Neurologic symptoms are three times more common in cases of bilateral subclavian steal compared with cases of unilateral disease [10].

Significant physical examination findings of patients with subclavian steal syndrome are more than 15 mmHg brachial systolic blood pressure differences in two arms (which obviously is absent in cases of bilateral subclavian stenosis), reduced and/or delayed upper extremity pulses, and subclavian or carotid bruit. Subclavian steal can also present with chest pain in patients that their internal mammary artery (IMA) has been used for coronary artery bypass graft surgery (CABG) and is known as coronary steal syndrome [11]. In the setting of hemodynamically significant stenosis in ipsilateral subclavian artery stenosis, flow through IMA may reverse or “steal” during upper extremity exercise and can cause chest pain for the patient. It should be noted that subclavian stenosis, the higher arm blood pressure is generally the nondiseased limb. In cases of severe bilateral and/or subclavian plus innominate stenosis, as in this case, the arm blood pressures are inaccurate, and ankle blood pressures are a more accurate reflection for the patient true blood pressure.

Subclavian steal syndrome diagnosis can be made by noninvasive imaging studies of upper extremity and cerebrovascular arterial system. In most experienced labs, duplex ultrasound can demonstrate stenosis of the subclavian artery and reversal of blood flow in vertebral arteries. Duplex can also assess the degree of stenosis throughout the remainder of the extracranial carotid system and assess the direction of blood flow in the vertebral arteries. Magnetic resonance imaging (MRI) and angiography (MRA) is alternative method for diagnostic workup and can give detailed information regarding the extracranial and intracranial vascular system. Computed tomography angiography and conventional angiography are other imaging modalities for diagnosis of subclavian steal syndrome.

Subclavian steal syndrome is often found in patient with concomitant cardiovascular diseases including lower-extremity peripheral artery disease and coronary artery disease. Secondary prevention and cardiovascular risk factor modification is the mainstay of treatment of subclavian stenosis and subclavian steal syndrome. These interventions include blood pressure control, cholesterol modification, glycemic control in diabetic patients, smoking cessation, lifestyle modifications, and antithrombotic/antiplatelet therapy. In symptomatic patients, treatment options include angioplasty, endarterectomy and patch aortosubclavian artery bypass or carotid-subclavian artery bypass surgery. Extrathoracic approach is the most popular surgical approach with overall patency rate of 95%, 86%, and 73% in one, three, and five years followup, respectively [12]. Although no randomized trials available, retrospective observational studies have shown equal effectiveness but fewer complication rates with angioplasty and/or stenting [13]. De Vries et al. in a retrospective observational study in 110 patients have shown more than 90% initial success rate with subclavian angioplasty with or without stenting [14]. In this study, significant restenosis (>70%) was observed in 7% at a median followup of 23 months. 

In summary subclavian steal syndrome is blood flow reversal in vertebral arteries usually caused by severe stenosis of the subclavian artery or innominate artery. The clinical presentation of subclavian steal syndrome is variable, ranging form asymptomatic to cerebrovascular ischemia. Subclavian stenosis is an independent risk factor for overall and cardiovascular mortality. Subclavian stenosis can be readily recognized by duplex ultrasound performed in experienced centers and with a protocol that includes imaging of the proximal subclavian arteries. Our vascular laboratory routinely assesses bilateral subclavian and innominate arteries as a part of the carotid artery duplex examination. Our patient presented with unrecognized hypertensive cardiomyopathy in the setting of falsely normal arm blood pressure reading due to severe stenosis of the innominate and left subclavian arteries. Surprisingly, she did not have any neurologic symptoms. In such a patient/situation, an intervention to the left subclavian artery to assess accurate blood pressure may be appropriate.
